# Impact of Exogenous Treatment with Histidine on Hepatocellular Carcinoma Cells

**DOI:** 10.3390/cancers14051205

**Published:** 2022-02-25

**Authors:** Yusun Park, Yeonju Han, Dongwoo Kim, Sua Cho, WonJin Kim, Hyemin Hwang, Hye Won Lee, Dai Hoon Han, Kyung Sik Kim, Mijin Yun, Misu Lee

**Affiliations:** 1 Division of Life Sciences, College of Life Science and Bioengineering, Incheon National University, Incheon 22012, Korea; pys803@inu.ac.kr (Y.P.); han0317@inu.ac.kr (Y.H.); lovelysua84@inu.ac.kr (S.C.); katips@inu.ac.kr (W.K.); tellso@inu.ac.kr (H.H.); 2 Department of Nuclear Medicine, Severance Hospital, Yonsei University College of Medicine, Seoul 03722, Korea; kdwoo@yuhs.ac; 3 Department of Internal Medicine, Severance Hospital, Yonsei University College of Medicine, Seoul 03722, Korea; lorry-lee@yuhs.ac; 4 Department of Surgery, Severance Hospital, Yonsei University College of Medicine, Seoul 03722, Korea; dhhan@yuhs.ac (D.H.H.); kskim88@yuhs.ac (K.S.K.); 5 Institute for New Drug Development, College of Life Science and Bioengineering, Incheon National University, Incheon 22012, Korea

**Keywords:** hepatocellular carcinoma, sorafenib, cancer metabolism, histidine, drug sensitivity

## Abstract

**Simple Summary:**

Sorafenib (Nexavar@) is the only currently approved anti-cancer drug for patients with advanced hepatocellular carcinoma (HCC). However, despite the development of strategies combining sorafenib with other cytotoxic chemotherapeutic agents to overcome sorafenib resistance, clinical trial results are still disappointing. In this study, we examined the enhancement of tumor responses to sorafenib by exogenous histidine treatment.

**Abstract:**

Hepatocellular carcinoma (HCC) is one of the leading causes of cancer-related deaths worldwide. Sorafenib, a multi-kinase inhibitor, is the first-line therapy for advanced HCC. However, long-term exposure to sorafenib often results in reduced sensitivity and the development of resistance. Although various amino acids have been shown to contribute to cancer initiation and progression, little is known about the effects of histidine, a dietary essential amino acid that is partially taken up via histidine/large neutral amino acid transporter (LAT1), on cancer cells. In this study, we evaluated the effects of histidine on HCC cells and sensitivity to sorafenib. Remarkably, we found that exogenous histidine treatment induced a reduction in the expression of tumor markers related to glycolysis (GLUT1 and HK2), inflammation (STAT3), angiogenesis (VEGFB and VEGFC), and stem cells (CD133). In addition, LAT1 expression was downregulated in HCC tumor regions with high expression of GLUT1, CD133, and pSTAT3, which are known to induce sorafenib resistance. Finally, we demonstrated that combined treatment with sorafenib and histidine could be a novel therapeutic strategy to enhance the sensitivity to sorafenib, thereby improving long-term survival in HCC.

## 1. Introduction

Hepatocellular carcinoma (HCC) is one of the leading causes of cancer-related deaths worldwide and commonly develops in patients with chronic liver inflammation caused by viral infections, metabolic syndrome, or high alcohol intake [1,2]. Since early-stage HCC is asymptomatic, more than 50% of patients are diagnosed at an intermediate or advanced stage [3]. Therefore, these patients are not eligible for surgical resection and liver transplantation, which can improve long-term survival in HCC [4].

Sorafenib (Nexavar@) is an oral multi-kinase inhibitor that targets tyrosine kinase receptors, thereby exerting anti-angiogenic and anti-proliferative effects [5]. According to new guidelines from the Asian-Pacific Association for the Study of the Liver (APASL) and the European Association for the Study of the Liver (EASL), sorafenib is recommend as the first-line treatment for patients with advanced HCC who are unsuitable for locoregional therapies [4,5,6,7]. Furthermore, the American Association for the Study of Liver Diseases (AASLD) also recommends sorafenib as a systemic agent [8]. However, the anticancer effect of sorafenib is short-lived due to a rapid reduction in drug sensitivity and the acquisition of resistance by tumor cells [4,5,9]. Generally, patients with HCC receiving sorafenib treatment acquire resistance within 6 months, and overall survival is increased by only 3 months [9]. Several mechanisms have been implicated in the low tumor-cell sensitivity to sorafenib, such as the tumor microenvironment, epithelial-to-mesenchymal transition, and cancer stem cells [10,11]. Hypoxia is caused by dysregulated vascularization and vigorous metabolic activity; it is a major tumor microenvironmental cause of sorafenib resistance in HCC via HIF1α and nuclear factor kappa B (NF-κB) activation [12,13,14]. The combination of sorafenib with HIF1α-targeted therapy or HIF1α inhibitors has been demonstrated to overcome sorafenib resistance in several HCC cell lines in vitro and in an in vivo animal model [14].

Cancer metabolism is an emerging hallmark of cancer. It is necessary to fulfill the bioenergetic and biosynthetic demands for tumor growth and adaptation to changing tumor microenvironments [15]. HCC is also characterized by significant alterations in metabolic processes, ranging from glucose metabolism and energy production to amino acid and fatty acid metabolism [16,17]. It is well known that increased glucose uptake in HCC is related to tumor aggressiveness and poor clinical outcomes [18,19,20]. However, high glycolytic activity is not universally observed across all HCC tumor types, and more commonly, heterogeneous metabolic phenotypes involve the differential utilization of nutrients and unregulated nutrient transport systems [21]. Recent studies have investigated the important roles of amino acids as alternative energy sources for cancer initiation and progression [22]. The metabolism of glutamine and serine is well reported in various cancers, but much less is known about other amino acids, such as histidine [23,24,25].

Studies of changes in cancer metabolism associated with sorafenib resistance have focused on central carbon metabolism, including glycolysis and the tricarboxylic acid cycle [25,26]. There are interesting reports of a novel role for extracellular histidine, which can increase the sensitivity of cancer cells to a chemotherapeutic agent used in the treatment of various solid tumors and blood cancers [27]. In this study, we investigated whether histidine could be used to overcome sorafenib resistance. In particular, we investigated the effect of histidine on HCC cells under hypoxic conditions. In addition, the expression levels of the histidine transport system and markers related to sorafenib resistance were explored using tumor tissues from patients with HCC. Finally, we evaluated the use of histidine and sorafenib as a combination therapy to enhance drug sensitivity.

## 2. Materials and Methods

### 2.1. Chemicals

Sorafenib (Santa Cruz (Dallas, TX, USA) was dissolved in dimethyl sulfoxide (DMSO) (Sigma Aldrich, St. Louis, MO, USA) to make a stock solution at concentration of 10 mM and stored at −80 °C. L-Histidine and D-glucose were purchased from Sigma-Aldrich. They were dissolved in pure water at 100 mM and 1 M, respectively.

### 2.2. Cell Culture and Treatment

HepG2 cells from the Korean Cell Line Bank (KCLB, Seoul, Korea) were cultured in RPMI-1640 (Gibco, Thermo Inc., Wilmington, DE, USA), and Hep3B cells from KCLB were cultured in Dulbecco’s modified Eagle’s medium (DMEM) (Gibco, Thermo Inc.) with 10% fetal bovine serum (FBS, Hyclone, Marlborough, MA, USA) and 1% penicillin−streptomycin (Gibco, Thermo Inc.). All cells were incubated in an incubator (37 °C, 5% CO_2_). We performed a regular mycoplasma test for contamination-free cells. A hypoxic chamber (Billups-Rothenberg, Inc., San Diego, CA, USA) under 1% O_2_, 5% CO_2_, and 94% N_2_ was used for generating a hypoxic condition. After 8 h of incubation, the cells were harvested for further analysis. To silence *SLC7A5*, HepG2 cells were transfected with a mixture of two si*SLC7A5* (Bioneer, Daejeon, Korea) using Lipofectamine RNAiMAX (Invitrogen, Carlsbad, CA, USA) following to the manufacturer’s instructions. Scrambled siRNA was used as the negative control. The sequences of siRNAs targeting human *SCL7A5* were as follows: (i) sense: 5′-CUGAUCGCCGUCUCCUUCUtt-3′; antisense: 5′-AGAAGGAGACGGCGAUCAGtt-3′; (ii) sense: 5-GUUUUGUGCUAACGUCUUAtt-3; antisense: 5′-UAAGACGUUAGCACAAAACtt-3′.

### 2.3. Western Blotting

To extract total protein, sodium dodecyl sulfate (SDS) lysis buffer (1% SDS, 60 mM Tris-HCl) with protease inhibitor (Roche, Basel, Switzerland) and phosphatase inhibitor (GenDEPOT, Katy, TX, USA) was used. WB was performed as previously reported [28]. The primary antibodies used were as follows: hexokinase 2 (HK2, ab209847, EPR20839, 1:500), glucose transporter 1(GLUT1, ab652, 1:2000), L-type amino acid transporter 1 (LAT1, ab208776, EPR17573, 1:1000), vascular endothelial growth factor B (VEGFB, ab110649, EPR4555, 1:1000), VEGFC (ab135506, 1:2000), CD133 (ab19898, 1:1000) and Snail/Slug (ab180714, 1:1000) from Abcam (Cambridge, UK), signal transducer and activator of transcription 3 (STAT3, #9139, 1:1000), pSTAT3 (#9145, D3A7, 1:2000) from Cell Signaling Technology (Danvers, MA, USA), and β-actin-HRP (sc-47778, C4, 1:5000) from Santa Cruz Biotechnology (Dalla, TX, USA). For the digital visualization of the chemiluminescent WB, the ChemiDoc XRS (Bio-Rad, Hercules, CA, USA) was used. These experiments were replicated at least three times with similar results. ImageJ (National Institutes of Health, Bethesda, MD, USA) and BioRad Image Lab 6 (Bio-Rad) were used for band quantification.

### 2.4. RNA Sequencing and Real-Time PCR (RT-PCR)

Total RNA was extracted by TRIzol reagent (Invitrogen). RNA was quantified using an ND-2000 spectrophotometer (Thermo Inc.). Total RNAs were extracted from HepG2 cells after histidine treatment under hypoxic conditions, as previously reported [29]. RNA sequencing and data analysis were assessed by EBIOGEN Inc (Seoul, Korea). For quantitative RT-PCR, SYBR^®^ Green Realtime PCR Master Mix (TOYOBO, Osaka, Japan) were used to generate cDNA and a C1000™ Thermal Cycler (Bio-Rad) was used to measure gene expression. Expression levels of mRNA were measured in triplicate for each experiment. Gene expression levels were normalized to mRNA expression levels of the housekeeping gene, beta-2 microglobulin (B2M) before statistical analysis. The following primers (Bioneer) were used: *HK2* (Forward 5′-AAGGTAGAAATGGAGCGAGGT-3′, reverse 5′- CCCGGAAATTTGTTCCTCCAA-3′), *SLC7A5* (Forward 5′-CCTCCATCCTCTCCATGATCC-3′, reverse 5′- AGCCAGTTGAAGAAGCTGAAG-3′), and *B2M* (Forward 5-TTACTCACGTCATCCAGCAGA-3′, reverse 5′-AGAAAGACCAGTCCTTGCTGA-3′).

### 2.5. Migration and Cell Viability Assay

Migration assays were performed using 24-well BioCoat (BD Biosciences, Heidelberg, Germany). HepG2 cells (2 × 10^5^ cells/well) in EBSS medium containing 1% FBS and sorafenib, histidine, or a combination of sorafenib and histidine were added to the insert. The lower well was filled with EBSS supplemented with 10% FBS. After 24 h, migrated cells were fixed in 4% paraformaldehyde (PFA) for 20 min and stained with 0.1% crystal violet for visualization. Three independent experiments were performed for these assays, with three technical replicates. Migrated cells were counted using ImageJ (National Institutes of Health, Bethesda, MD, USA). Cell viability was measured after sorafenib, or a combination of sorafenib and 800 μM histidine treatment by WST-1 colorimetric assays (Roche, Mannheim, Germany). Six technical replicates were performed in three independent experiments. For the colony-formation assay, HepG2 cells (1000 cells/well) were plated onto a 6-well plate and treated with sorafenib, histidine, or a combination of sorafenib and histidine in EBSS medium. After 24 h, the medium was changed every 3–4 days for 3 weeks. Cells were fixed by 4% PFA and stained with crystal violet (0.1% *v*/*v* in PBS). Three independent experiments were performed for these assays, with three technical replicates. Colony counting were performed using ImageJ (National Institutes of Health, Bethesda, MD, USA).

### 2.6. Immunostaining

All animal experiments were approved by The Institutional Animal Care and Use Committee of Incheon University (approval number: 2019-13). Female BALB/c nude mice, 4–5 weeks-old) were purchased from DBL (Seoul, Korea). HepG2 and Hep3B cells (1 × 10^6^ cells) mixed with Matrigel (100 µL, Sigma Aldrich) were subcutaneously injected into BALB/c nude mice. When the tumor size reached 1000 mm^3^, the mice were euthanized by cardiac puncture and tumors were fixed with 10% formaldehyde (Sigma Aldrich). Thirty-seven human HCC tissues, obtained from twenty-nine patients, were used in this study. The characteristics of patients in the study are summarized in Table S1. This human study was approved by the Institutional Review Board of Yonsei University Severance Hospital (Seoul, Korea). All experiments were performed as per the relevant guidelines and regulations (Yonsei IRB number: 4-2015-0904). All patients provided oral and written consent after receiving detailed information on the study and agreed to data collection. Using paraffin slides from human patients and animals, immunostaining was performed as previously described [30]. The primary antibodies used were: pSTAT3 (#9145; Cell Signaling Technology; dilution 1:300), LAT1 (ab208776; Abcam; 1:300), GLUT1(ab115730; Abcam;1:500), CD133 (ab19898; Abcam;1:2000), and HK2 (ab104836; Abcam; 1:500). Images were acquired using an Olympus BX53 microscope, and the positive tumor regions were quantified using Olympus Cell Sens (Olympus, Tokyo, Japan).

### 2.7. Statistical Analysis

GraphPad Prism (GraphPad Software Inc., San Diego, CA, USA) was used for statistical analysis. Data were analyzed by unpaired *t*-tests or one-way analysis of variance (ANOVA) with Tukey’s multiple comparisons tests. Pearson’s correlation coefficient was used to calculate the correlation between the two factors. All data are expressed as means  ±  SD (standard deviation) and statistical significance was set at *p* < 0.05.

## 3. Results

### 3.1. Effect of Exogenous Hist Idine Treatment on HepG2 Cells

**Figure 1:**
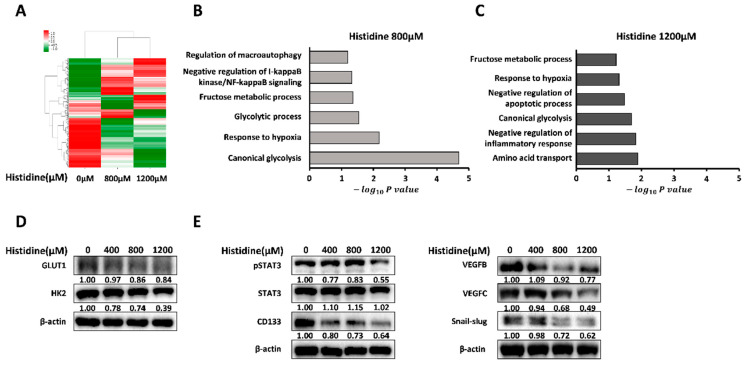
Effect of histidine in HepG2 cells at the gene and protein levels. (**A**) A heatmap of RNA-seq data was generated to show relative transcriptomic differences. Fold change of the indicated genes obtained by gene expression profiling of HepG2 cells after treatment with 0 μM, 800 μM, and 1200 μM histidine in EBSS under hypoxic conditions. (**B**,**C**) Summary of DAVID functional annotation analysis to assess GO enrichment for differentially expressed genes in HepG2 cells after 800 μM (**B**) and 1200 μM (**C**) versus 0 μM histidine treatment. (**D**) HepG2 cells were incubated with indicated concentrations of histidine in EBSS media under hypoxia (1% oxygen). After 24 h, GLUT1, hexokinase2 (HK2), and β-actin expression levels were examined by Western blotting. The ratios of the band intensities normalized by actin are reported below the respective panels. The presented images are cropped and the whole blot images are presented in Figure S6. Data shown represent one of three independent experiments with similar results. The quantification of band intensities from three independent experiments are shown in Figure S2A,B. (**E**) In the same conditions as in (**D**), the protein levels of pSTAT3, STAT3, VEGFB, VEGFC, CD133, Snail/slug, and β-actin were analyzed by Western blotting. The ratios of the band intensities normalized by actin are reported below the respective panels. The presented images are cropped and the whole blot images are presented in Figure S7. Data shown represent one of three independent experiments with similar results. The quantification of band intensities from three independent experiments are shown in Figure S2C–G.

A hypoxic microenvironment leads to sorafenib resistance in HCC. To investigate the effect of histidine on sorafenib resistance, global gene expression profiles were assessed using HepG2 cells after extracellular histidine treatment under hypoxic conditions (Figure 1A and Figure S1A,B). In total, 729 and 869 genes were dysregulated in HepG2 cells after histidine treatment at 800 μM and 1200 μM, respectively, compared to levels in untreated cells (fold change vs. 0 µM histidine > 1.5, *p* < 0.05). To identify the biological significance of the dysregulated genes, DAVID GO online analysis tool (https://david.ncifcrf.gov/tools.jsp, accessed on 30 November 2021) were used. Dysregulated genes after histidine treatment were enriched for GO terms related to the glycolytic process, glycolysis, inflammation, and hypoxia (Figure 1B,C). To confirm the results of the gene expression analysis, we performed Western blotting for proteins encoded by genes included in the GO analysis. The expression levels of glycolysis-related proteins GLUT1 and HK2 were downregulated in a dose-dependent manner after histidine treatment (Figure 1D). Reduced levels of phosphorylated STAT (pSTAT), a key factor in inflammation, were also observed. Additionally, the expression levels of VEGFB and VEGFC (angiogenesis marker), CD133 (stem cell marker), and Snail/slug (metastasis marker) were downregulated after histidine treatment (Figure 1E). Altogether, histidine treatment induced an anti-tumor effect in HCC cells.

### 3.2. Expression of SLC7A5/LAT1 in HCC Cell Lines with Differences in Glycolysis

**Figure 2:**
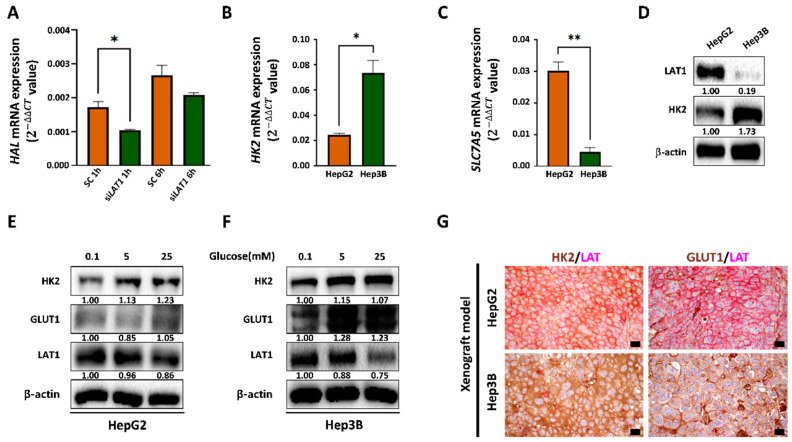
Comparison of the expression of LAT1 in HCC cells with differences in glycolytic activity. (**A**) Hep3 cells were transfected with scrambled siRNA (SC) or siRNA targeting LAT1 (si*LAT1*) in HepG2 cells. After 24 h of incubation, cells were treated with histidine (1200 μM) in EBSS media under hypoxia (1% oxygen) for an additional 1h and 6h, respectively. The mRNA levels of HAL were evaluated by quantitative RT-PCR. The results were normalized to B2M, a housekeeping gene, and evaluated by the 2−ΔΔCt method. Data are shown as the mean of three independent experiments ± SD. Statistical analyses were performed using GraphPad Prism and comparisons between groups were made using unpaired *t*-tests. * *p* < 0.05. (**B**,**C**) mRNA expression levels of *HK2* and *SLC7A5* were assessed by RT-PCR using probe sets specific for *HK2* (**B**) and *SLC7A5* (**C**) in HepG2 and Hpe3B. The expression of the target genes was normalized to that of B2M (housekeeping gene) using the 2^−ΔΔCt^ method. Data are shown as the mean of three independent experiments ± SD. Statistical analyses were performed using GraphPad Prism and comparisons between groups were made using unpaired *t*-tests. * *p* < 0.05; ** *p* < 0.01. (**D**) The expression levels of LAT1, HK2, and β-actin of HepG2 cells and Hep3B cells were evaluated by Western blotting. The ratios of the band intensities normalized by actin are reported below the respective panels. The presented images are cropped and the whole blot images are presented in Figure S8. Data shown represent one of three independent experiments with similar results. The quantification of band intensities from three independent experiments are shown in Figure S3A,B. (**E**) HepG2 after incubation with the indicated concentration of glucose for 8 h. The expression levels of HK2, GLUT1, LAT1, and β-actin were measured by Western blotting. The ratios of the band intensities normalized by actin are reported below the respective panels. The presented images are cropped and the whole blot images are presented in Figure S9. Data shown represent one of three independent experiments with similar results. The quantification of band intensities from three independent experiments are shown in Figure S3C–E. Quantitation of the signals obtained by densitometry was in Figures S3C–E and S8. (**F**) Hep3B after incubation with the indicated concentration of glucose for 8 h. The expression levels of HK2, GLUT1, LAT1, and β-actin were measured by Western blotting. The ratios of the band intensities normalized by actin are reported below the respective panels. The presented images are cropped and the whole blot images are presented in Figure S10. Data shown represent one of three independent experiments with similar results. The quantification of band intensities from three independent experiments are shown in Figure S3C–E. (**G**) Formalin-fixed, paraffin-embedded tumor tissues from HepG2- and Hep3B-xenograft models were used. Double staining was performed using GLUT1, HK2, and LAT1 antibodies followed by counterstaining with hematoxylin. GLUT1 and HK2 are shown in brown and LAT1 is indicated by magenta pink. Scale bars: 20 μm.

Since histidine treatment downregulated glycolytic markers, we further evaluated whether the utilization of histidine is associated with glycolysis in HCC cells. *SLC7A5*/LAT1 belongs to the APC superfamily and mediates the influx of neutral essential amino acids, including histidine, into cells [31]. A decrease in histidine ammonia−lyase histidine (HAL) expression was found in HepG2 cells with *LAT1* knockdown after histidine treatment, suggesting that extracellular histidine uptake is mediated in part by LAT1 (Figure 2A). Next, *SLC7A5*/LAT1 expression was evaluated in HCC cell lines with differences in glycolysis at the gene and protein levels. HepG2 cells with lower expression of *HK2*/HK2 showed higher expression levels of *SLC7A5*/LAT1 than those in Hep3B cells (Figure 2B–D). Remarkably, LAT1 expression was downregulated after glucose-induced glycolysis in HCC cells (Figure 2E,F and Figure S3C–E). Finally, we generated xenograft models with HepG2 and Hep3B cells and performed double-immunostaining of HK2 (brown) and LAT1 (magenta pink) as well as GLUT1 (brown) and LAT1 (magenta pink). HCC tissues in the HepG2-xenograft model with low expression of HK2 and GLUT1 showed higher expression of LAT1 compared with that in the Hep3B-xenograft model with high expression of HK2 and GLUT1 (Figure 2G). Thus, LAT1 expression is dysregulated in glycolytic HCC cells.

### 3.3. Expression of SLC7A5/LAT1 in Patients with HCC

**Figure 3:**
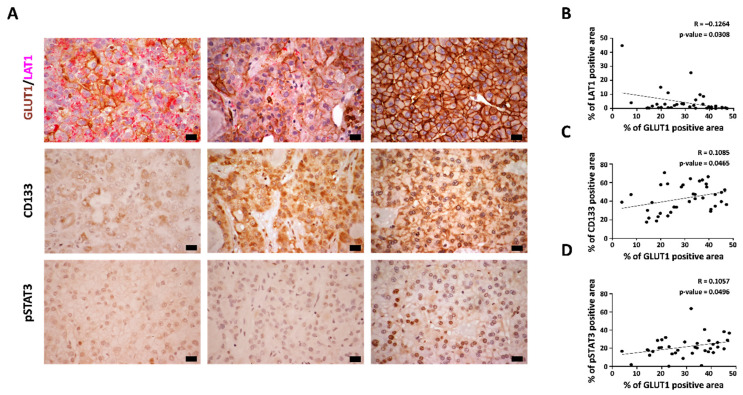
Expression levels of LAT1, GLUT1, CD133, and pSATA3 in human HCC tumor tissues. (**A**) Formalin-fixed, paraffin-embedded liver tissues from human patients with HCC were used. Double staining immunohistochemistry was conducted using GLUT1 and LAT1 antibodies. GLUT1 is indicated in brown and LAT1 is magenta pink. Single immunohistochemistry was also conducted using CD133 and pSTAT3 antibodies and counterstaining with hematoxylin. Scale bars: 20 μm. (**B**) Correlation between the LAT1-positive area and GLUT1-positive area. (**C**) Correlation between the GLUT1-positive area and CD133-positive area. (**D**) Correlation between the GLUT1-positive area and pSTAT3-positive area. Pearson’s correlation coefficient was used to calculate the correlation between the two factors. Scale bars: 20 μm.

To confirm our findings, LAT1 expression was assessed in human HCC tumor tissues (*n* = 37). Similar to the results obtained using a cell line and animal model, tumor regions with decreased expression of GLUT1 (brown) showed increased expression of LAT1 (magenta pink) and vice versa (Figure 3A). Quantitative analyses revealed a negative correlation between LAT1- and GLUT1-positive areas (r = −0.1264, *p* = 0.0308) (Figure 3B). We also assessed the expression levels of sorafenib-resistant resistance-related proteins, such as CD133 and pSTAT3. There were positive correlations between CD133 and GLUT1 expression (r = 0.1085, *p* = 0.0465) (Figure 3C) and between pSTAT3 and GLUT1 expression (r = 0.1057, *p* = 0.0496) (Figure 3D). Altogether, LAT1 expression was downregulated in HCC tumor regions with high expression of GLUT1, CD133, and pSTAT3, which are known to induce sorafenib resistance.

### 3.4. Increased Anti-Cancer Effect after Combined Treatment with Sorafenib and Histidine

**Figure 4:**
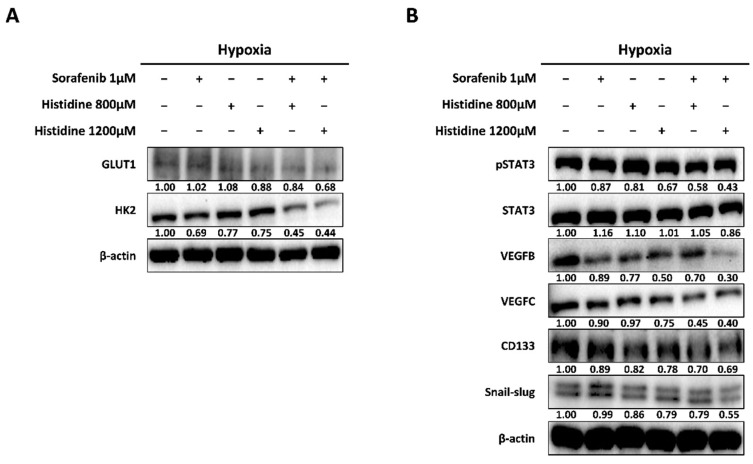
Anti-cancer effect of the combination of sorafenib and histidine. (**A**) HepG2 cells were treated with the combination of sorafenib and histidine at the indicated concentrations in EBSS media under hypoxia (1% oxygen). After 24 h, GLUT1, LAT1, HK2, and β-actin expression levels were determined by Western blotting. The ratios of the band intensities normalized by actin are reported below the respective panels. The presented images are cropped and the whole blot images are presented in Figure S11. Data shown represent one of three independent experiments with similar results. The quantification of band intensities from three independent experiments are shown in Figure S4A,B. (**B**) In the same conditions as used for (A), pSTAT3, STAT3, VEGF B, VEGF C, CD133, Snail/slug, and β-actin expression levels were measured by Western blotting. The ratios of the band intensities normalized by actin are reported below the respective panels. The presented images are cropped and the whole blot images are presented in Figure S12. Data shown represent one of three independent experiments with similar results. The quantification of band intensities from three independent experiments are shown in Figure S4C–G.

**Figure 5:**
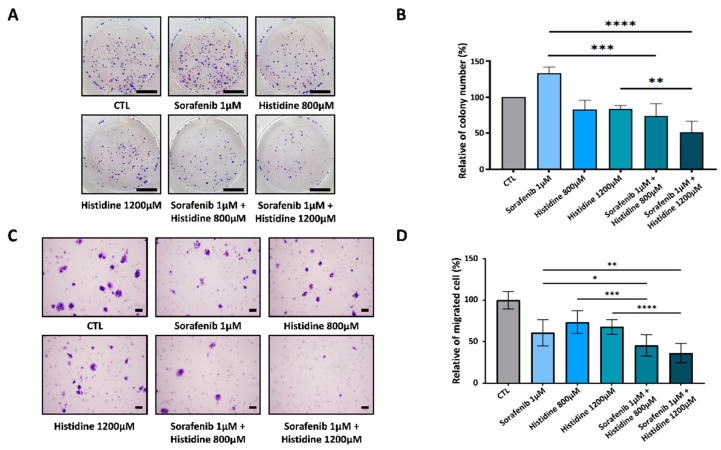
The anti-cancer effect of the combined treatment with sorafenib and histidine. (**A**) A colony-formation assay was performed in HepG2 cells treated the combination of 1 µM sorafenib and 800 μM or 1200 μM L-histidine in EBSS media under hypoxia (1% oxygen) for 24 h. After treatment, cells were changed to normal growth media. After 3–4 weeks, cells were fixed and stained with crystal violet. Scale bars: 1 cm (**B**) Quantitative analyses were performed to determine relative colony numbers. Data are shown as the mean of three independent experiments ± SD. Statistical analyses were performed using GraphPad Prism and comparisons between groups were made using ANOVA with Tukey’s multiple comparisons tests. * *p* < 0.05; ** *p* < 0.01; *** *p* < 0.001; **** *p* < 0.0001. (**C**) A migration assay was conducted with HepG2 cells. Inserted HepG2 cells were treated with the indicated condition. After 24 h incubation under hypoxia, cells were fixed and stained with crystal violet. Scale bars: 20 μm (**D**) Bar graphs show relative migrated cells Data are shown as the mean of three independent experiments ± SD. Statistical analyses were performed using GraphPad Prism and comparisons between groups were made using ANOVA with Tukey’s multiple comparisons tests. * *p* < 0.05; ** *p* < 0.01; *** *p* < 0.001; **** *p* < 0.0001.

Sorafenib-resistant HCC cells exhibit glycolytic, mesenchymal, and stemness features [11]. To investigate whether extracellular histidine treatment could enhance the anti-cancer effect of sorafenib, the combination of histidine with sorafenib was used to treat HepG2 cells. The expression levels of the glycolysis-related proteins GLUT1 and HK2 were reduced after the combined treatment (Figure 4A). We also observed the downregulation of pSTAT3, VEGF, CD133, and Snail/slug in HepG2 cells after the combined treatment (Figure 4B). To evaluate the synergistic anti-cancer effects of histidine and sorafenib, we conducted cell-viability and colony-formation assays in HepG2 cells. The reduction in cell viability after the combined treatment with sorafenib and histidine was higher compared to that after the single sorafenib treatment (Figure S5). The colony number was higher after the single treatment with 1 µM sorafenib than in the control (+33% vs. control) due to the formation of sorafenib-resistant cells. After combined treatment with histidine and sorafenib, the colony number was reduced compared with that after each single treatment (−60% vs. sorafenib treatment) (Figure 5A,B). Next, to confirm the increased anti-cancer effect of the combined treatment, we also measured cell migration after treatment with sorafenib and histidine (Figure 5C). The number of migrated HepG2 cells was lower after the combined treatment than after treatment with a single agent (Figure 5D). Thus, histidine exhibited anti-tumor effects following sorafenib treatment in HepG2 cells.

## 4. Discussion

Despite the development of strategies combining sorafenib with other cytotoxic chemotherapeutic agents to overcome sorafenib resistance, clinical trial results are still disappointing. Our results revealed that extracellular histidine suppressed the expression levels of tumorigenesis and of tumor-progression-related genes and proteins in HCC cells. Furthermore, we investigated the synergistic effect of histidine and sorafenib on proliferation and migration, suggesting that extracellular histidine has the potential to improve the treatment efficacy of sorafenib in HCC.

Histidine is a dietary essential amino acid and is degraded by a conserved enzymatic pathway that begins with the conversion of histidine to urocanate via HAL [32]. LAT1, a sodium-independent transporter, mediates the transport of neutral essential amino acids into cells. Thus, to investigate the role of extracellular histidine via LAT1 in HCC cells, all experiments were performed in a nutrient-deficient environment using EBSS. The identification of histidine as a high-affinity substrate for LAT1 has been conducted in various in vitro cellular systems [31]. In our study, reduced HAL expression after histidine treatment was detected in *SLC7A5*-silenced HCC cells, indirectly showing that the intake of histidine is mediated by LAT1. Remarkably, the expression of LAT1 increased following reduced glucose concentrations. In line with our observations, Ryo et al. reported that the transcriptional activity of the *SLC7A5* promoter is increased after culturing under glucose-free conditions in endothelial cells [33]. In addition, the inhibition of glucose metabolism evoked LAT1 upregulation in leukemia cells [34]. Thus, the upregulation of LAT1 may be associated with glucose intake and utilization in tumor cells. Indeed, there is a negative correlation between GLUT1 and LAT1 expression in the tumor regions of human HCC.

Few studies have reported the role of histidine in cancer cells. Clair et al. demonstrated that histidine inhibits cell motility induced by tumor cell motility factors in ovarian and melanoma tumor cell lines [35]. Yan et al. also reported that histidine significantly diminished cytokine release following liver injury [36]. Notably, another study has shown that methotrexate, an antifolate chemotherapeutic agent administered in combination with histidine, reduces tumorigenesis in a mouse model of leukemia [27]. These findings led us to investigate the potential therapeutic value of histidine in HCC. In the present study, histidine supplementation in HCC cells reduced the expression of tumor markers related to glycolysis (GLUT1 and HK2), inflammation (pSTAT3), angiogenesis (VEGFB and VEGFC), stem cells (CD133), metastasis (Snail/slug), and cell migration. Moreover, the combination of sorafenib and histidine resulted in greater reductions in these tumor markers compared with those for single treatment, suggesting a synergistic antitumor effect of histidine. Among these tumor markers, increased CD133 expression is mediated by the activation of STAT3 signaling, which promotes HCC progression and induces a poor response to sorafenib [37]. Indeed, we also detected a positive correlation between CD133 and GLUT1 and between pSTAT3 and GLUT1 in tumors from patients with HCC. Similar to our results, the combination of sorafenib with STAT3 inhibitors, such as nifuroxazide, has been shown to enhance the cytotoxicity of sorafenib in an HCC xenograft animal model [38].

Changes in cancer cell metabolism by heterogeneous nutrient utilization in cancer cells can serve to evaluate tumor responses to medical interventions and tumor development [39]. Our global gene expression analysis after histidine treatment in HCC cells revealed the dysregulation of genes involved in glycolysis and amino acid transport, suggesting that histidine treatment exerts metabolic changes. However, the mechanism by which histidine alters metabolic properties of cancer cells remains unclear and should be elucidated in future studies.

## 5. Conclusions

We provide the first evidence for the antitumor effect of histidine in HCC cells. In addition, our results showed that the anticancer effect of sorafenib is improved when administered in combination with histidine. Clinically, histidine supplementation appears to be relatively safe in both animals and humans [40]. Thus, exogenous histidine treatment could be a novel therapeutic strategy for patients with sorafenib-resistant HCC.

## Data Availability

The data presented in this study are available on request from the corresponding author.

## Supplementary Materials

The following supporting information can be downloaded at: https://www.mdpi.com/article/10.3390/cancers14051205/s1, Table S1: The characteristics of the 29 patients with hepatocellular carcinoma (HCC). Figure S1: The scatter plot of global gene expression in HepG2 cells after 800 μM (A) and 1200 μM (B) versus 0 μM histidine treatment. Figure S2: Quantification of Western blot bands relative expression in Figure 1. Figure S3: Quantification of Western blot bands relative expression in Figure 2. Figure S4: Quantification of Western blot bands relative expression in Figure 4. Figure S5: Cell viability in HepG2 cells after the combined treatment with sorafenib and histidine. Figure S6: Whole blot images of Figure 1D. Figure S7: Whole blot images of Figure 1E. Figure S8: Whole blot images of Figure 2D. Figure S9: Whole blot images of Figure 2E. Figure S10: Whole blot images of Figure 2F. Figure S11: Whole blot images of Figure 4A. Figure S12: Whole blot images of Figure 4A.
