# Supplementary material for: Impact of Exogenous Treatment with Histidine on Hepatocellular Carcinoma Cells
Source: Cancers (Basel). 2022 Feb 25;14(5):1205. doi: 10.3390/cancers14051205 (PMC8909034; doi:10.3390/cancers14051205)
Supplement: Supplementary file 1 [file cancers-14-01205-s001.zip › cancers-1559080-supplementary.pdf]

**Characteristics**	
**Age, year**	
**Mean ± SD**	57.4±10.01
**Range**	39-74
**Sex**	
**Male**	72.4%
**Female**	29.6%
**Tumor size (cm)**	
**Mean ± SD**	3.43±1.18
**Range**	1.7-5.6
**AFP (ng/ml)**	
**Mean ± SD**	357.06±758.7
**Range**	1.47-3150.32

Table S1. The characteristics of 29 patients with HCC.

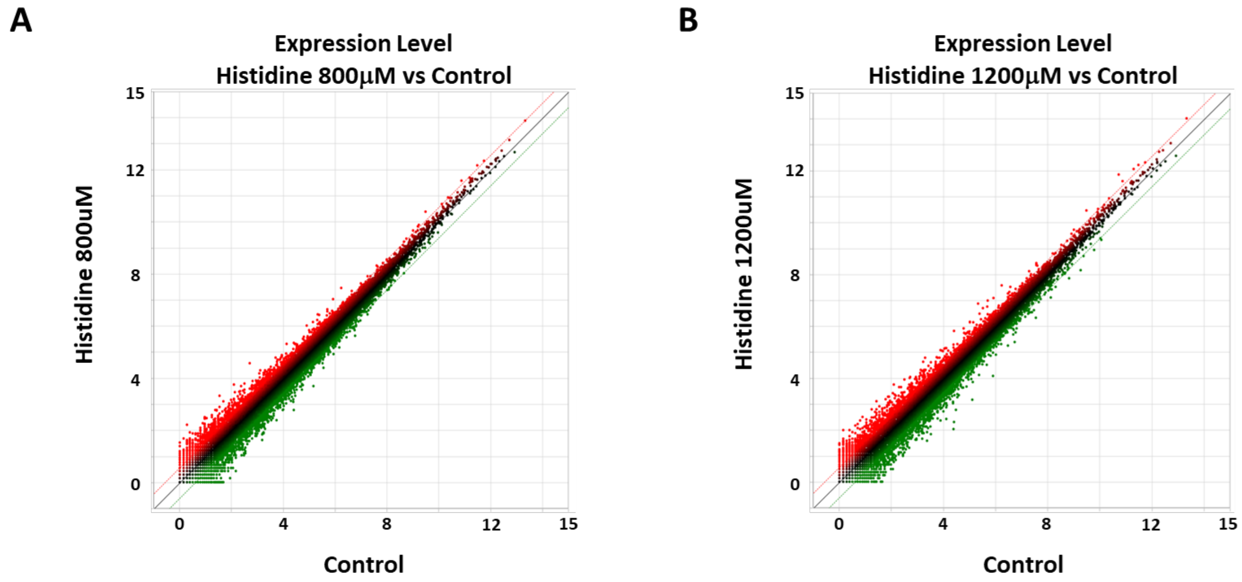

Figure S1. The scatter plot of global gene expression in HepG2 cells after 800 μM (A) and 1200 μM (B) versus 0 μM histidine treatment.

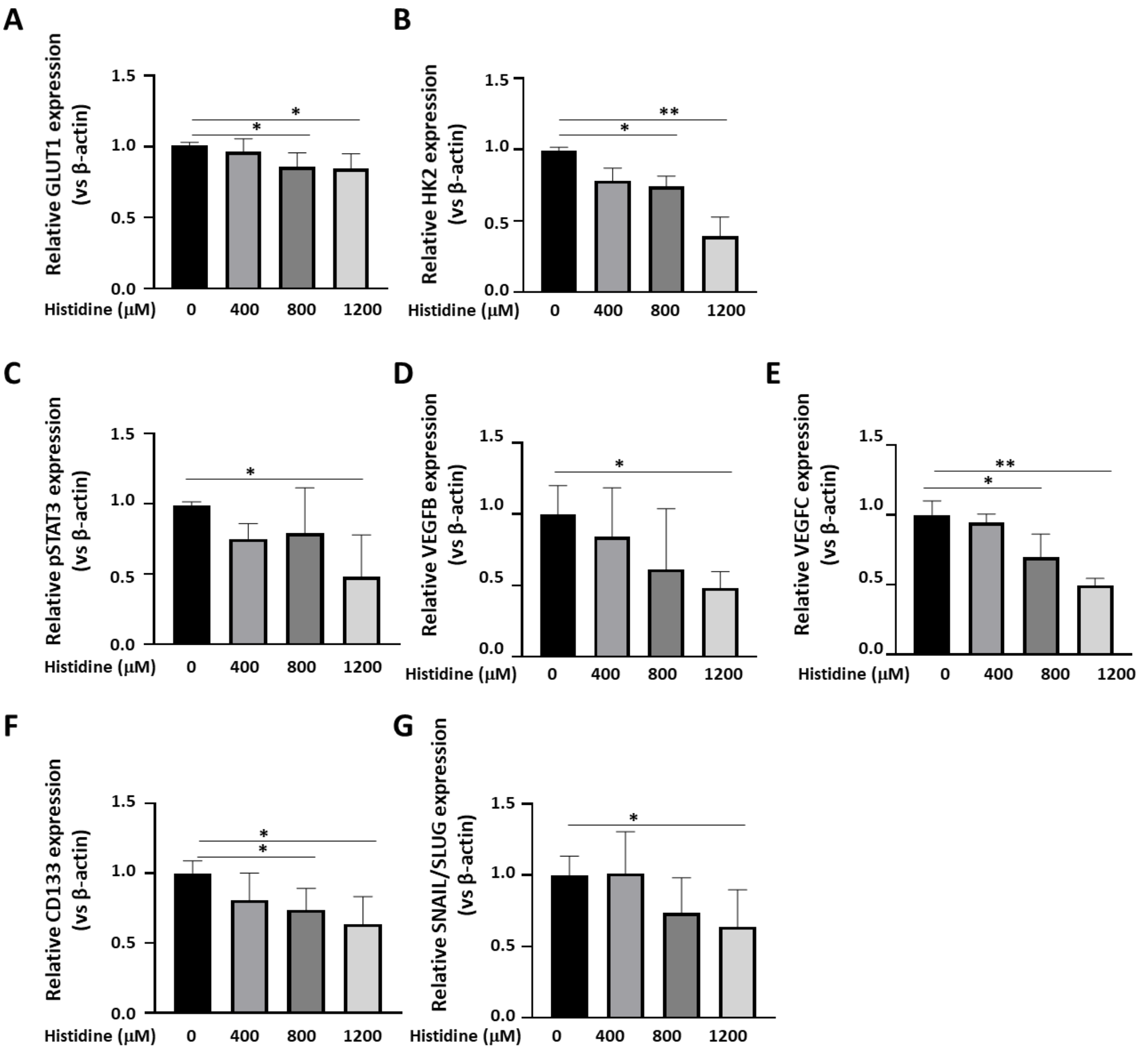

Figure S2. Densitometry intensity ratio of each band in Fig 1 from replicated WB (n=3). (A,B) Densitometry intensity ratio of GLUT1 and HK2 in Figure 1D from three independent WB. (C-G) Densitometry intensity ratio of pSTAT3, VEGFB, VEGFC, CD133 and Snail/slug in Figure 1E from three independent WB (*; $p < 0.05$; **; $p < 0.01$).

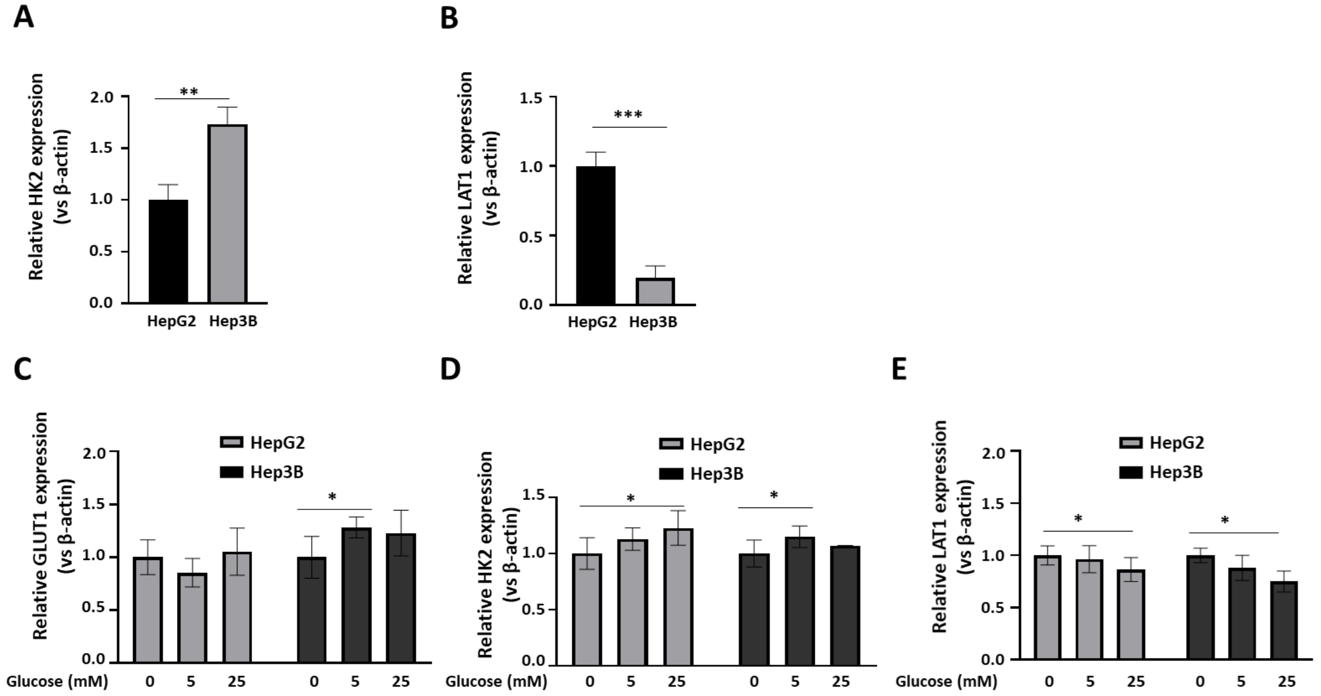

Figure S3. Densitometry intensity ratio of each band in Figure 2 from replicated WB (n=3). (A, B) Densitometry intensity ratio of HK2 and LAT1 in Figure 2D from three independent WB. (C-E) Densitometry intensity ratio of HK2, GLUT1 and LAT1 in Figure 2E and 2F from three independent WB (*; $p < 0.05$; **; $p < 0.01$; ***; $p < 0.001$).

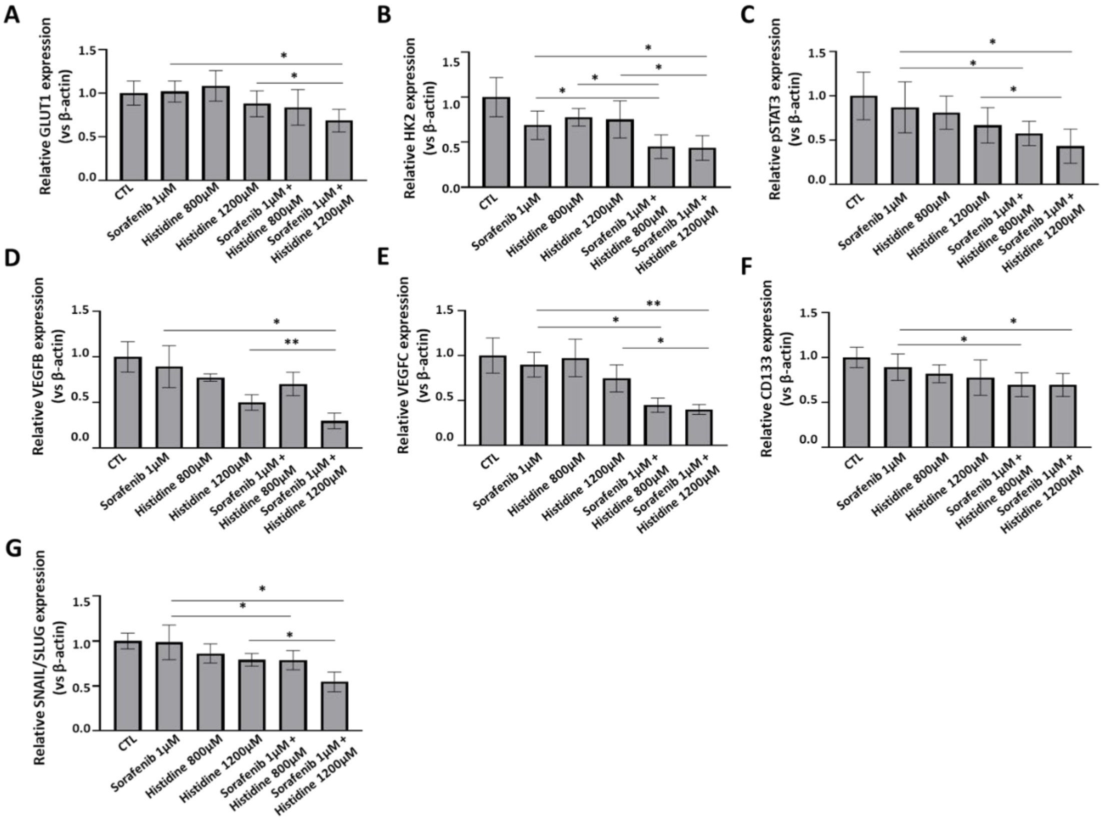

Figure S4. Densitometry intensity ratio of each band in Figure 4 from replicated WB (n=3). (A, B) Densitometry intensity ratio of GLUT1, HK2 and LAT1 in Figure 4A from three independent WB. (C-G) Densitometry intensity ratio of pSTAT3, VEGFB, VEGFC, CD133 and snail/slug in Figure 4B from three independent WB (*; $p < 0.05$; **; $p < 0.01$).

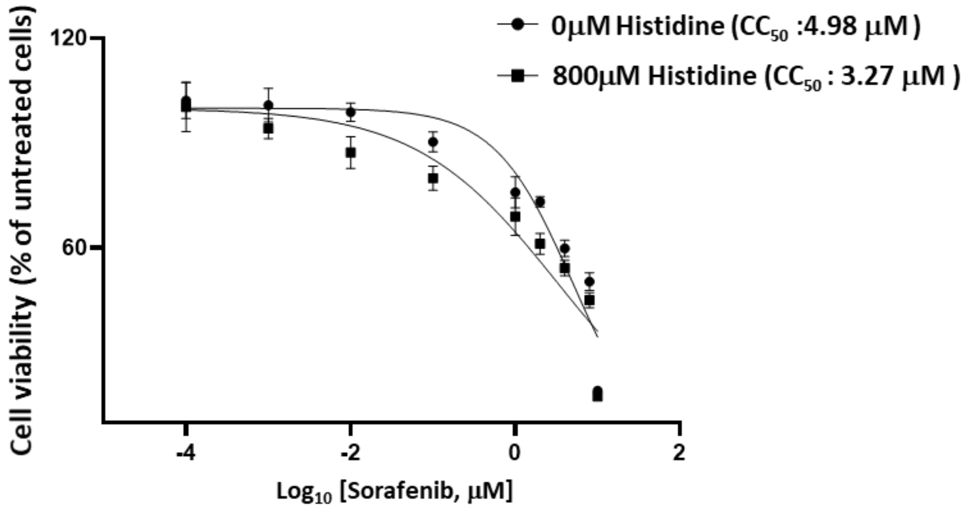

Figure S5. Cell viability in HepG2 cells after combined treatment with sorafenib and histidine. Cell viability of HepG2 cells after the indicated treatment for 24 h under hypoxic conditions. Data are shown as the mean of three independent experiments ± SD. The 50% cytotoxic concentration (CC_{50}) was calculated for the indicated treatment by nonlinear regression analysis using GraphPad Prism.

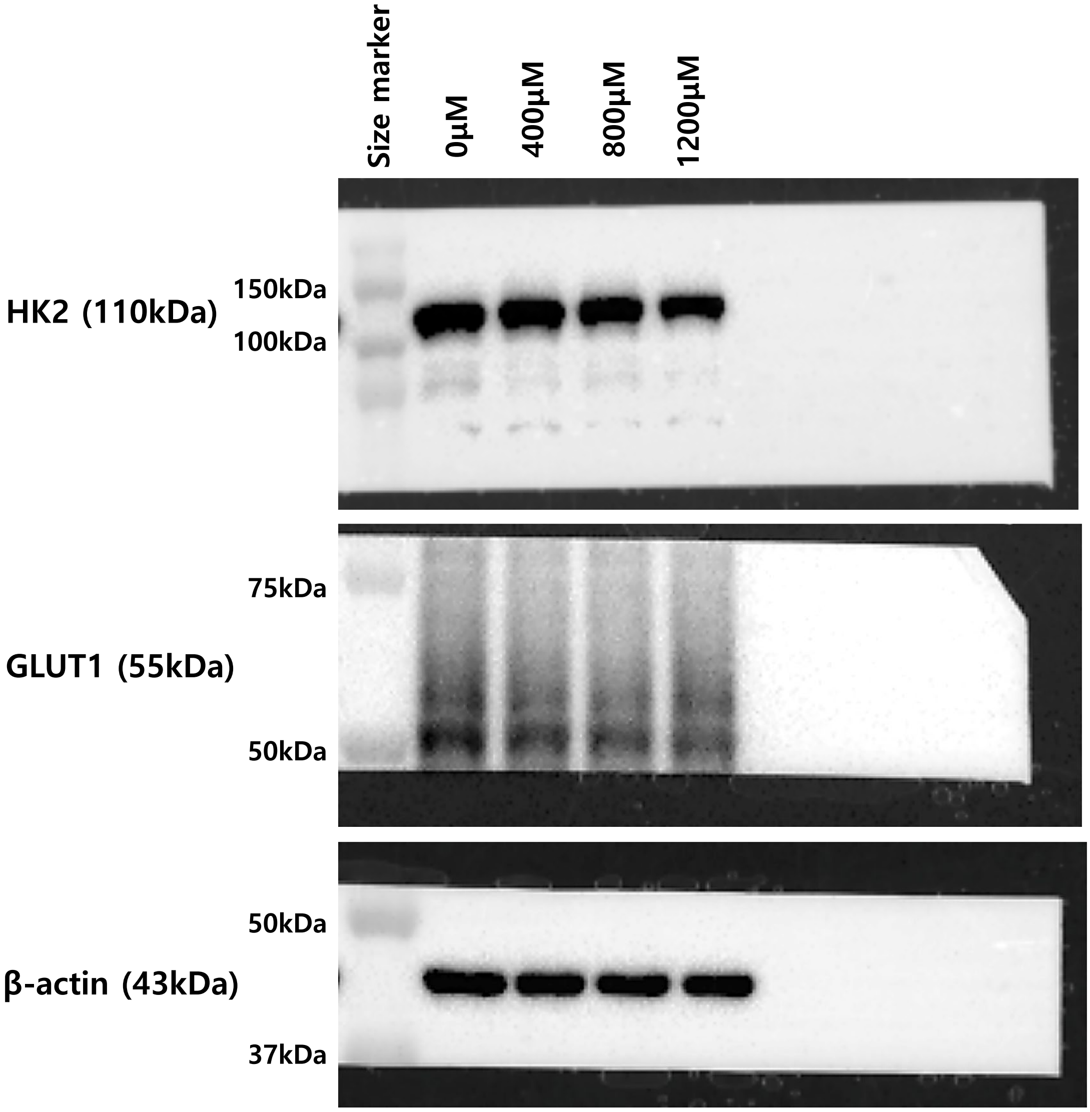

	0µM	400µM	800µM	1200µM
HK2/β-actin	1.240506252	1.01388949	0.957504406	0.554450224
GLUT1/β-actin	0.911231279	0.881179816	0.780532083	0.767815642

Figure S6. Whole blot images and the band intensity of Figure 1D.

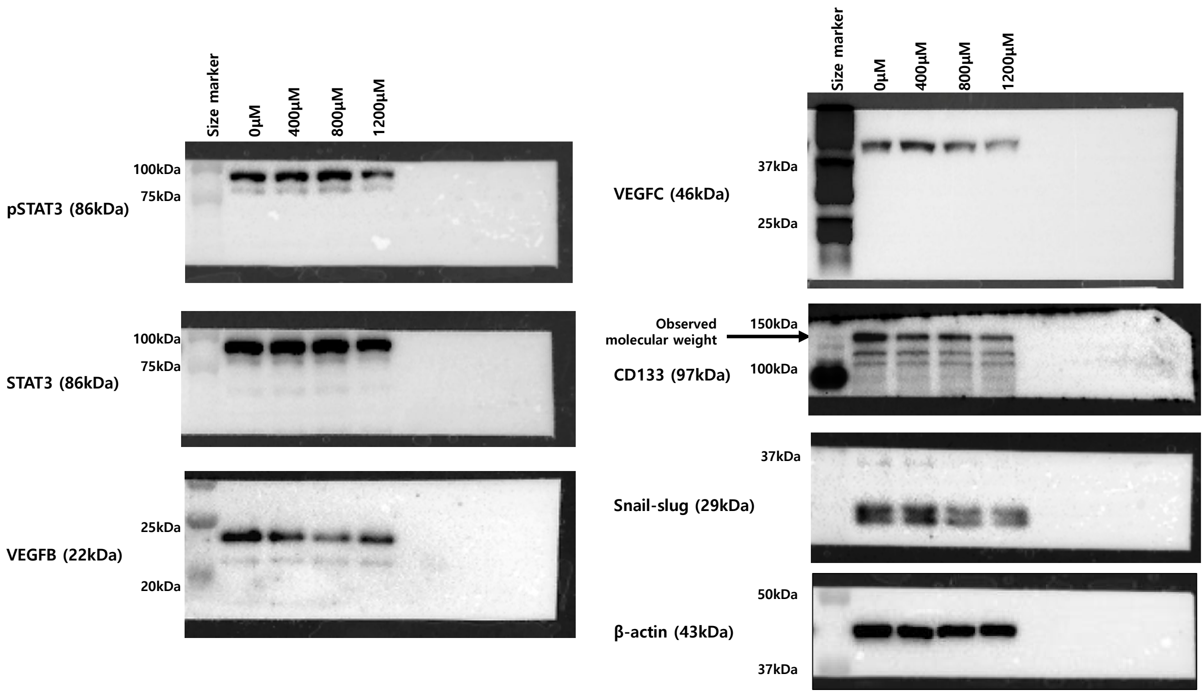

	0μM	400μM	800μM	1200μM
**pSTAT3/β-actin**	0.645808126	0.506772855	0.537655659	0.322282443
**STAT3/β-actin**	0.921362078	1.011892197	1.058046628	0.939849154
**VEGFB/β-actin**	0.707965418	0.637349018	0.48739076	0.454533569
**VEGFC/β-actin**	0.906921894	0.850132925	0.615495963	0.445004567
**CD133/β-actin**	0.945506391	0.757341979	0.690985986	0.600734712
**Snail-slug/β-actin**	0.849326633	0.834072409	0.608238073	0.523429628

Figure S7. Whole blot images and the band intensity of Figure 1E.

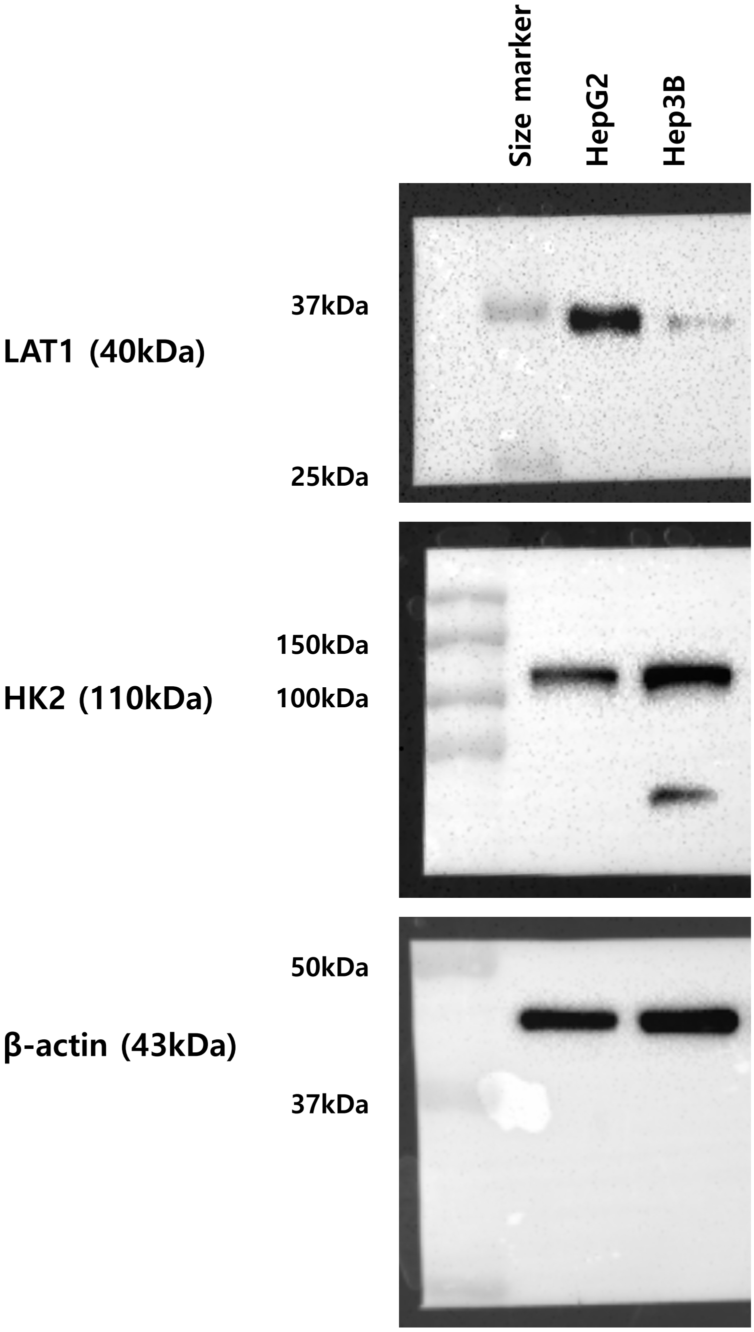

	HepG2	Hep3B
**LAT1/β-actin**	1.052932255	0.19779284
**HK2/β-actin**	0.928665681	0.974479995

Figure S8. Whole blot images and the band intensity of Figure 2D.

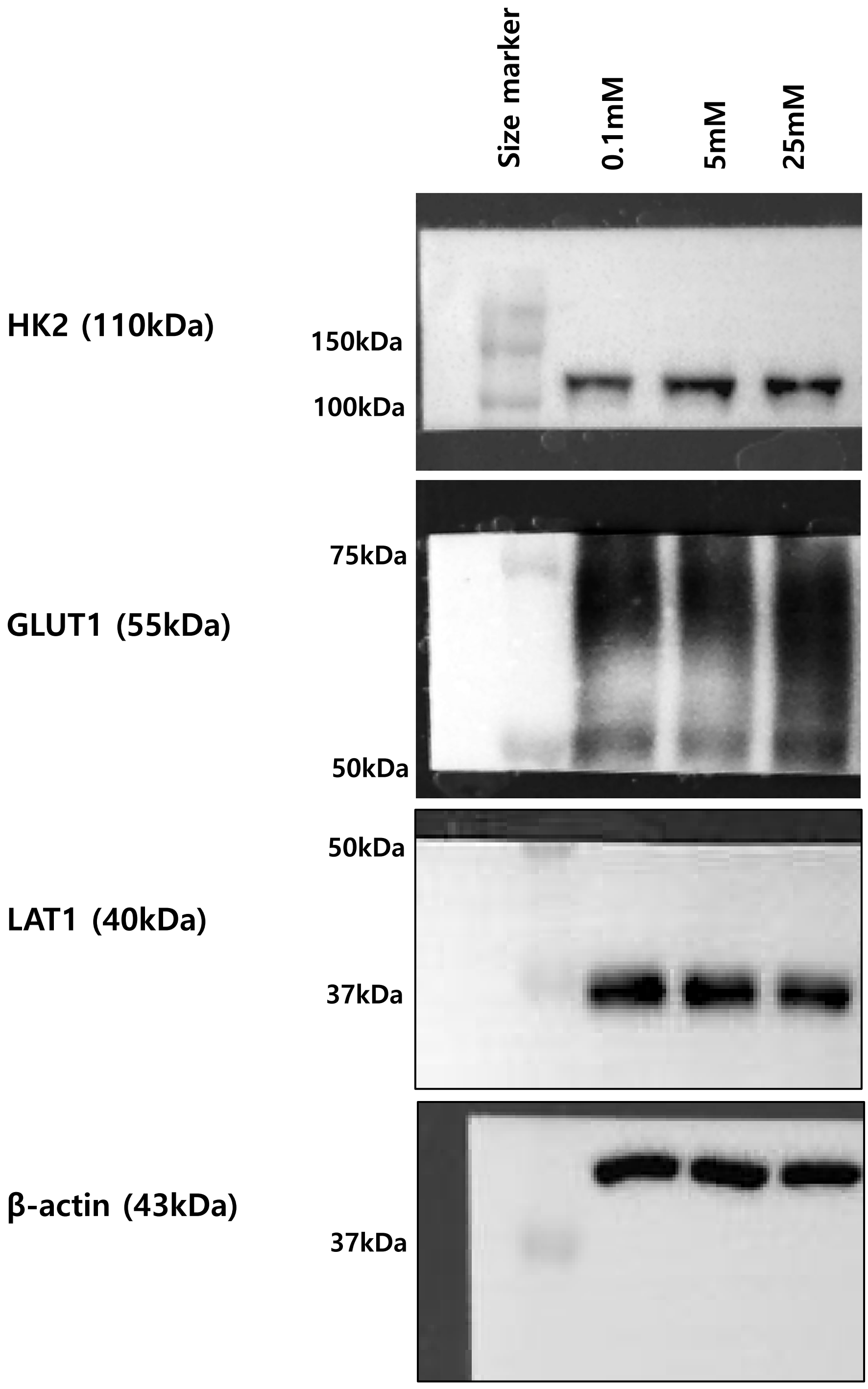

	0.1mM	5mM	25mM
HK2/β-actin	0.718256057	0.809715146	0.881158497
GLUT1/β-actin	0.721342643	0.616741536	0.759904836
LAT1/β-actin	0.815044248	0.785452034	0.703886854

Figure S9. Whole blot images and the band intensity of Figure 2E.

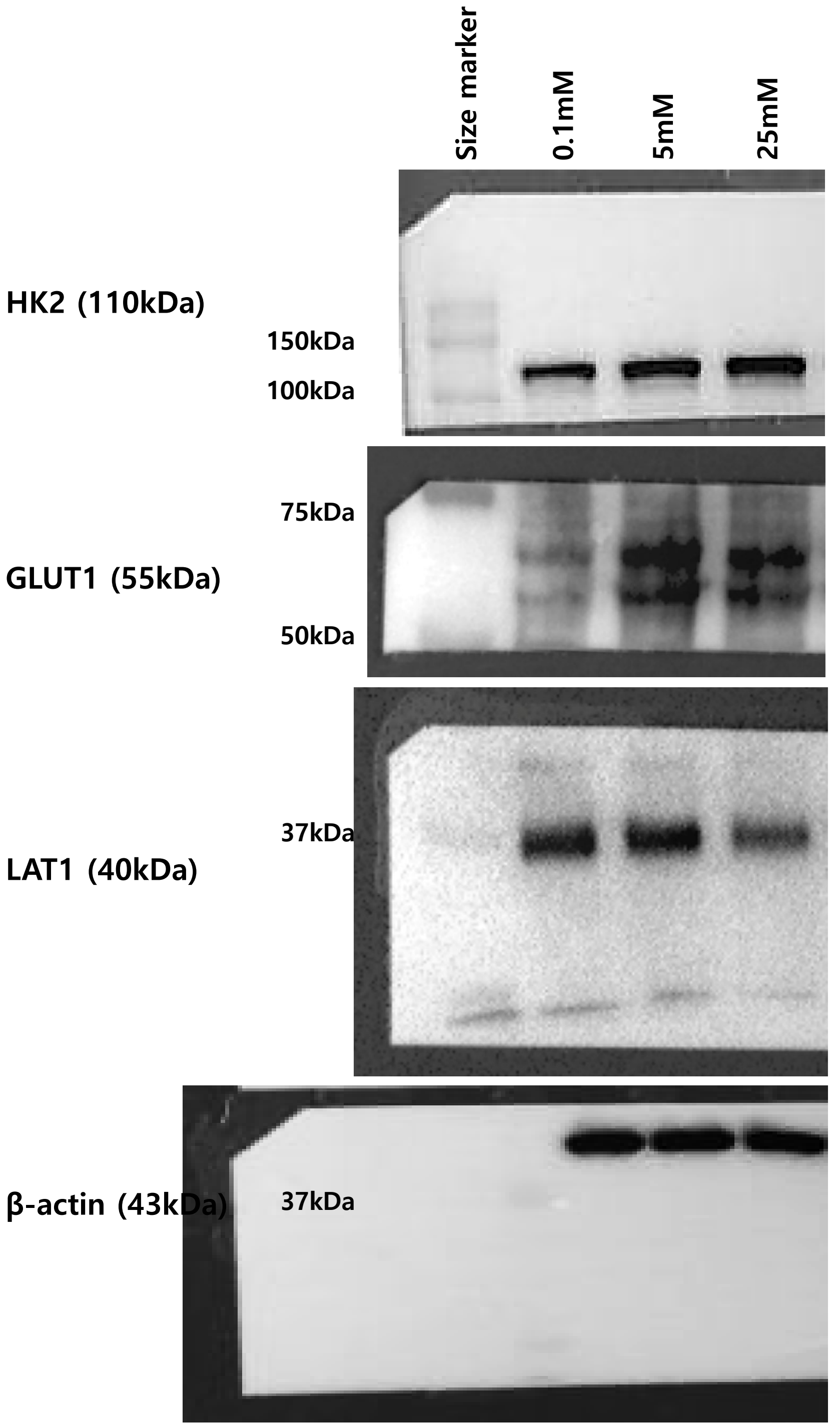

	0.1mM	5mM	25mM
HK2/β-actin	0.479636111	0.55081031	0.511463131
GLUT1/β-actin	0.662398854	0.849042534	0.814152769
LAT1/β-actin	0.519966211	0.457593221	0.390374511

Figure S10. Whole blot images and the band intensity of Figure 2F.

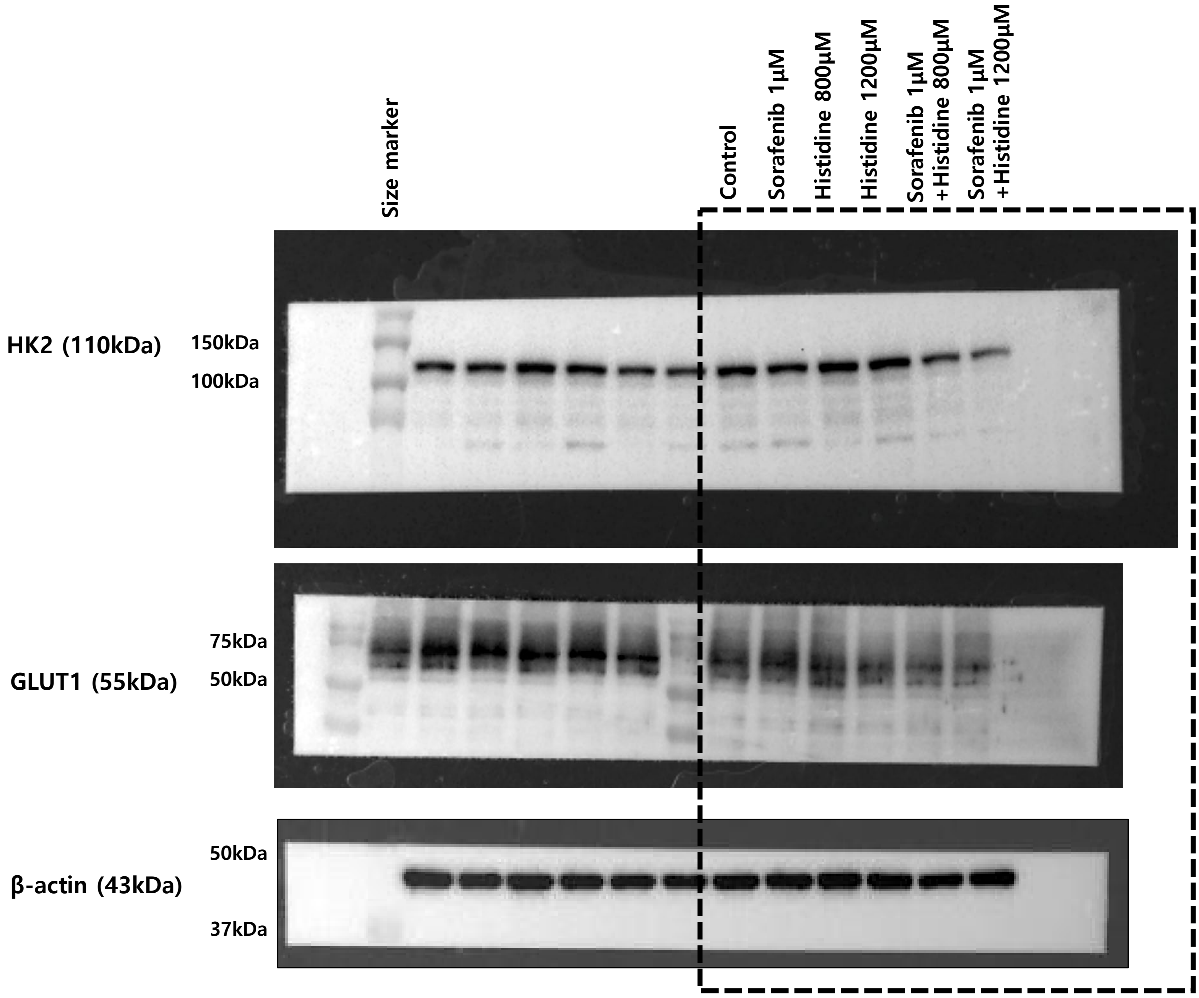

	Control	Sorafenib 1μM	Histidine 800μM	Histidine 1200μM	Sorafenib 1μM + Histidine 800μM	Sorafenib 1μM + Histidine 1200μM
HK2/ β-actin	0.883494329	0.608231117	0.672539052	0.682299029	0.403810359	0.406938662
GLUT1/ β-actin	0.863208664	0.878519835	0.934852308	0.758272514	0.722325462	0.5906945

Figure S11. Whole blot images of Figure 4A.

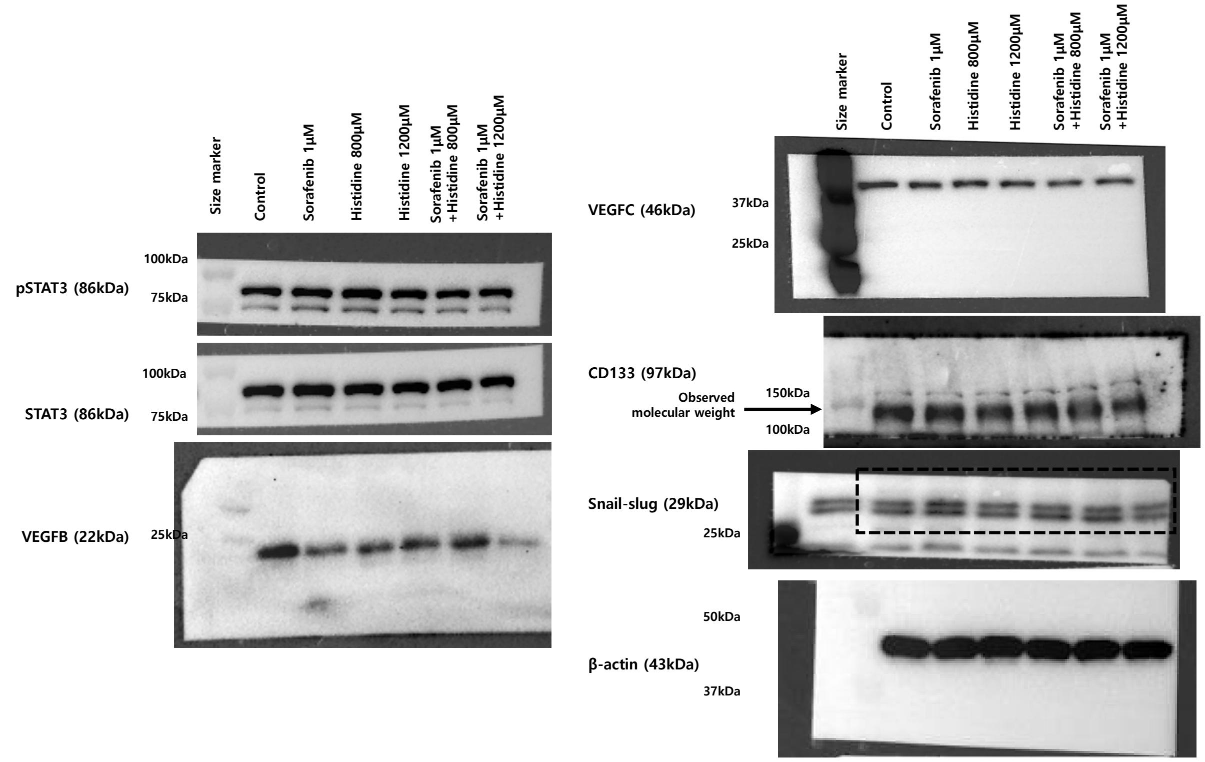

	Control	Sorafenib 1μM	Histidine 800μM	Histidine 1200μM	Sorafenib 1μM + Histidine 800μM	Sorafenib 1μM + Histidine 1200μM
pSTAT3/ β-actin	0.92675442	0.851572988	0.789712821	0.663721001	0.525821255	0.435958225
STAT3/ β-actin	0.820864661	0.952812671	0.901801945	0.83005884	0.859496729	0.708914816
VEGFB/ β-actin	0.839947558	0.719978537	0.555868357	0.383203024	0.470573246	0.205591109
VEGFC/ β-actin	0.519664122	0.467193701	0.505702461	0.388267203	0.233880345	0.208831138
CD133/ β-actin	0.755376701	0.674225185	0.619033587	0.586538255	0.527213973	0.5246275
Snail-slug/ β-actin	0.727217444	0.716904133	0.627147239	0.575402337	0.572845864	0.39701405

Figure S12. Whole blot images of Figure 4A.
